# Safety and effectiveness of biphasic insulin aspart 30/70 (NovoMix® 30) when switching from human premix insulin in patients with type 2 diabetes: subgroup analysis from the 6-month IMPROVE™ observational study

**DOI:** 10.1111/j.1742-1241.2009.02012.x

**Published:** 2009-04

**Authors:** S Shah, M Benroubi, V Borzi, J Gumprecht, R Kawamori, J Shaban, M Shestakova, Y Wenying, P Valensi

**Affiliations:** 1 Department of Endocrinology, BhatiaS. L. Raheja HospitalMumbai, India; 2 Diabetes Centre, General Hospital of Athens “POLYKLINICI”Athens, Greece; 3 Department of Internal Medicine, Vittorio Emanuele HospitalCatania, Italy; 4 Department of Internal Diseases, Silesian School of MedicineZabrze, Poland; 5 Department of Metabolism and Endocrinology, Juntendo University School of MedicineTokyo, Japan; 6 Endocrinology and Metabolism, Windsor Regional Hospital, WindsorON, Canada; 7 Institute of Diabetes, Federal Scientific Centre of EndocrinologyMoscow, Russia; 8 Department of Endocrinology, China-Japan Friendship HospitalBeijing, China; 9 Department of Endocrinology-Diabetology-Nutrition, Jean Verdier Hospital, AP-HP, Paris Nord University, CRNH-IdF, Bondy CedexFrance

## Abstract

**Aims:**

IMPROVE™ is an open-label, multinational, non-randomised, 26-week observational study designed to evaluate the safety and effectiveness of biphasic insulin aspart 30 (BIAsp 30) in routine clinical practice. Here, we report data for patients switching to BIAsp 30 from human premixed insulin.

**Methods:**

Patients (*n*=3856) with type 2 diabetes previously receiving human premixed insulin with or without oral antidiabetic drugs were eligible for inclusion. Demographic data, efficacy end-points (HbA_1c_, fasting blood glucose and postprandial blood glucose) and safety end-points (serious adverse drug reactions, hypoglycaemia and adverse events) were collected at baseline and final visit. A subgroup analysis of mean dose change was also undertaken.

**Results:**

Switching patients to BIAsp 30 resulted in significant improvements in glycaemic control combined with a reduced risk of hypoglycaemia. Patients who reached the HbA_1c_ target (< 7%) had shorter diabetes duration, lower HbA_1c_ at baseline and needed less insulin. Over 30% of patients were able to reach this target without experiencing hypoglycaemia over the 26-week period. Compared with asymmetric dose switching, unit-for-unit switching resulted in the highest proportion of patients reaching HbA_1c_ target and incurred the least amount of dose titration.

**Conclusions:**

A unit-for-unit switch is the most effective as well as the simplest approach when transferring patients from biphasic human insulin 30 to BIAsp 30.

**What’s known**

- Premixed insulins provide a viable option for the treatment of type 2 diabetes.
- Premixed insulin analogues are able to mimic more closely the physiological insulin needs than human premixes.

**What’s new**

- IMPROVE™ is a multinational, open-label, observational study of biphasic insulin aspart 30 (BIAsp 30) treatment in type 2 diabetes in routine clinical practice.
- Guidelines for switching patients from human insulin premix to BIAsp 30 may be useful to physicians wishing to benefit from the advantages of a premixed analogue regimen.

## Introduction

Treatment regimens comprising premixed insulin are an established treatment option when starting insulin in type 2 diabetes patients (1). Indeed, insulin therapy is recommended by authorities such as the International Diabetes Federation (2) and American Association of Clinical Endocrinologists (3) in treatment initiation regimens, especially where HbA_1c_ levels are high (> 10%).

Human premixed insulin, often known as biphasic human insulin 30 (BHI 30), contains a fixed soluble human insulin component (making up 30% of the formulation) and neutral protamine Hagedorn (NPH) insulin (the remaining 70%). The soluble component, when injected 30 min before a meal, aims to lower postprandial glucose excursions, while NPH provides basal insulin coverage. Together, they can lower glycaemia and provide good glycaemic control in patients with type 2 diabetes (4,5). Human premixed insulin is, however, associated with relatively high risks of hypoglycaemia, probably owing to the mismatch between its pharmacokinetic profile and the physiological need (6).

Premixed insulin analogues, such as biphasic insulin aspart 30/70 (BIAsp 30) and lispro mix 25 (Mix 25) are, on the other hand, associated with a pharmacokinetic profile that more closely mimics insulin needs (7,8). As a result, better postprandial glucose control has been seen compared with human insulin premixes (5,8). Indeed, when used in an insulin initiation regimen in a 3-month, single-centre comparative study, BIAsp 30 was shown to achieve significantly better HbA_1c_ levels than human premix at the end of the study (9).

Data from randomised controlled trials involving premixed insulin analogues have consistently shown that these insulins can significantly lower HbA_1c_ levels and that they are an effective treatment for patients with type 2 diabetes (8,10–13). In addition, a large observational study (PRESENT) – using BIAsp 30 in routine clinical practice – suggested that patients who transferred from human premix to BIAsp 30 for 6 months, with little increase in dose, significantly improved their glycaemic control and the rate of hypoglycaemia also decreased over time (14). In summary, there is an increasing body of evidence to support the use of premix insulin analogues over human premix insulin, but there is little evidence and practical guidance on how this transfer should be made. Indeed, it is likely that patients receiving human premix are using it with a combination of different oral antidiabetic drugs (OADs) and that the range of doses varies between patients.

The IMPROVE™ study is also an international, non-interventional, observational study carried out to investigate the safety profile and effectiveness of BIAsp 30 in the treatment of type 2 diabetes. In this subanalysis of the total cohort, data are reported for patients previously using BHI 30 who switched to BIAsp 30 at the same or lower dose and those who upgraded to a higher dose.

## Methods and patients

The study was performed in accordance with the Declaration of Helsinki and approval was gained from local ethics committees. All participants gave written informed consent.

Details of the study design of IMPROVE™ have been published elsewhere (15). In brief, IMPROVE™ was a 26-week, open-label, non-randomised, multicentre observational study in 11 countries (Canada, China, Greece, Gulf region, India, Iran, Italy, Japan, Poland, Russia and South Korea). Participating physicians received remuneration for the time spent registering patient data in accordance with local rules and regulations. Patients with type 2 diabetes being prescribed BIAsp 30 by their physician in routine clinical practice were eligible for inclusion into the study, but data from the subanalysis reported here only includes those patients who were previously receiving BHI 30 with or without (±) OADs.

BIAsp 30 (±OADs) was prescribed by the physician as part of routine treatment and administered once, twice or three times daily, depending on the patient’s needs; the dosage was also adjusted individually, as required, and information about the dose was recorded at baseline, 3 months and at the final visit (after 6 months).

The primary end-point was the incidence of major hypoglycaemic events reported as a serious adverse drug reaction (SADR) during the 26-week BIAsp 30 treatment period. Secondary end-points included further safety parameters: additional SADRs, adverse drug reactions and number of major and minor hypoglycaemic events (daytime and nocturnal). Major hypoglycaemic events were defined as events with symptoms consistent with hypoglycaemia in which the subject was unable to treat him/herself and that had one of the following characteristics: (i) blood glucose measurement < 2.8 mmol/l (< 50 mg/dl) or (ii) reversal of symptoms after either glucagon or intravenous glucose administration. Minor hypoglycaemic events were defined as events with either symptoms of hypoglycaemia that resolved with oral carbohydrate intake, glucagon or intravenous glucose or any symptomatic or asymptomatic blood glucose < 2.8 mmol/l (< 50 mg/dl). Finally, nocturnal hypoglycaemic events were classed as symptomatic events consistent with hypoglycaemia that occurred while sleeping, between bedtime after the evening insulin injection and before getting up in the morning [before morning determination of fasting blood glucose (FBG) and morning injection]. Major hypoglycaemic events were recorded over 13 weeks before each visit, while minor hypoglycaemic events were recorded over 4 weeks before each visit. These data were calculated as events per patient year.

Any changes in weight (kg) and body mass index (BMI, kg/m^2^) were calculated from recorded data. Measurements of effectiveness were recorded as additional secondary end-points, including HbA_1c_ (%), proportions of patients reaching targets of HbA_1c_ < 7.0%, FBG (mmol/l), postprandial blood glucose (PPBG; mmol/l) after all main meals. Finally, patient treatment satisfaction was recorded at the start and end of treatment using the DiabMedSat questionnaire (16).

The full analysis set was defined as all patients with a baseline visit and who had been prescribed BIAsp 30 at least once. The efficacy analysis set was defined as above, but only included patients who also had one measurement of hypoglycaemic event, blood glucose, weight or HbA_1c_ at baseline and final visit. Patient data presented in this manuscript are for those who had values for end-points at both the baseline and the end of study visits.

### Statistical methods

Statistical comparisons of BIAsp 30 end-points at baseline (week 0) and final visit (approximately week 26) were performed with paired *t*-tests for continuous variables, and with Wilcoxon signed-rank tests for discrete variables. Influence of predictor variables on the change in outcome variables was evaluated with ANCOVA models for continuous outcome variables and logistic models for discrete outcome variables. All testing used two-sided tests with the criteria set at α = 0.05.

## Results

### Patients

Baseline demographic data are shown in Table 1. The majority of patients (*n*=3413/3856, 83.3%) injected BHI 30 twice a day prestudy, and over half of patients in this subanalysis received at least two OADs. In the analyses that follow, overall cohort data have been presented, and in addition, patients have been stratified according to dose, i.e. whether they switched from BHI 30 to BIAsp 30 unit-for-unit or to a lower dose (< 90% of BHI dose) or a higher dose (> 10% dose increase).

**Table 1:** Patient demographics and prestudy therapy details

Demographic	Patients previously receiving human premix ± OADs (*n*=3856)
Age (years)	57.0 ± 11.5
Gender, male/female (%)	2230/1623 (57.9/42.1%)
BMI (kg/m^2^)	26.3 ± 4.9
Duration of diabetes (years)	10.7 ± 6.9
**Prestudy BHI 30 dose**	
lU	33.4 ± 17.8
lU/kg	0.49 ± 0.24
Percentage of patients injecting BHI 30 once/twice/three/four times daily prestudy (%)	11.4/84.0/4.4/0.2
Percentage of patients receiving 1, 2, > 2 OADs prestudy (%)*	26/38/16

Data are mean ± SD unless stated otherwise.

* OAD information missing for 20% of patients. BMI, body mass index; BHI, biphasic human insulin 30; OAD, oral antidiabetic drug.

### Effectiveness

Mean overall HbA_1c_ reduction from baseline was 1.84% at final visit (Table 2). Switching to BIAsp 30 from a human premix insulin regimen facilitated mean reductions in all measured indices of glycaemic control after 6 months of treatment: HbA_1c_ (20% reduction), FBG (34% reduction) and PPBG (33% reduction) were all improved (Table 2).

**Table 2:** Change from baseline in safety and effectiveness parameters when using biphasic insulin aspart 30 for 6 months

Effectiveness parameter	Baseline	3-month follow up	Final visit at 6 months	Change from baseline to final visit	p-value
HbA_1c_, %	9.21 ± 1.71	7.85 ± 1.18	7.37 ± 1.24	−1.84 ± 1.63	< 0.0001
FBG, mmol/l	10.29 ± 3.05	7.71 ± 1.96	6.81 ± 1.62	−3.48 ± 2.98	< 0.0001
PPBG breakfast, mmol/l	14.91 ± 4.04	10.92 ± 2.75	9.42 ± 2.32	−5.48 ± 4.04	< 0.0001
PPBG lunch, mmol/l	14.75 ± 4.61	10.42 ± 2.73	9.57 ± 2.55	−5.17 ± 4.41	< 0.0001
PPBG dinner, mmol/l	11.53 ± 3.70	8.95 ± 2.01	8.29 ± 1.79	−3.24 ± 4.10	< 0.0001
Major hypoglycaemia (events/patient/year)	0.355	n/a	0.028	0.331	< 0.0001
Minor hypoglycaemia (events/patient/year)	7.725	1.907	2.025	5.700	< 0.0001
Nocturnal minor hypoglycaemia (events/patient/year)	2.578	0.411	0.408	2.170	< 0.0001
Treatment satisfaction, % patients very/extremely satisfied	16.10%	n/a	56.90%	n/a	< 0.0001

Values obtained at the 3-month follow-up visit are also provided. Values are mean ± SD. Data from patients with values for each timepoint. FBG, fasting blood glucose; PPBG, postprandial glucose. Main cohort (*n*=3856).

At baseline, 3405 patients had an HbA_1c_≥ 7.0%, and 253 patients (6.3%) had an HbA_1c_ < 7.0% (data were missing for 198 patients). By the end of the study, 1489 patients (40.5%) achieved an HbA_1c_ < 7.0%. The demographics and outcome parameters for patients who achieved an HbA_1c_ < 7.0 and ≥ 7.0% at final visit are shown in Table 3. Patients who reached this target had significantly lower BMI and duration of diabetes than those who did not reach this target (BMI: 25.9 vs. 26.4 kg/m^2^, respectively, p = 0.0077; duration of diabetes: 9.9 vs. 11.3 years respectively, p < 0.0001; Table 3). Glycaemic control at baseline was also significantly better in patients who reached HbA_1c_ < 7.0% compared with those whose final HbA_1c_ was ≥ 7.0% (HbA_1c_: 8.47 vs. 9.71%, respectively, p < 0.0001; FBG: 9.84 vs. 10.59 mmol/l, p < 0.0001; PPBG: 14.20 vs. 15.49 mmol/l, p < 0.0001; Table 3).

**Table 3:** Demographics and safety and effectiveness outcomes for subjects who had HbA_1c_≥ 7.0% and < 7.0% at final visit

Study measure (*n*=3856)	HbA_1c_≥ 7% (*n*=2186)	HbA_1c_ < 7% (*n*=1489)	p-value for between groups
Age (years)	56.9 ± 11.28	57.1 ± 11.49	0.727
Gender, male/female (%)	1269/9.7 (58.1/41.9)	843/644 (56.7/43.3)	0.413
BMI (kg/m^2^)	26.4 ± 4.8	25.9 ± 4.4	0.0077
Duration of diabetes (years)	11.3 ± 6.8	9.9 ± 6.6	< 0.0001
Duration of prestudy insulin therapy (years)	2.9 ± 3.3	2.5 ± 3.3	< 0.0001
Percentage of patients injecting BHI 30 once/twice/three/four times daily prestudy (%)	11.7/85.2/3.2	10.6/83.9/5.4	0.002
**HbA_1c_, %**			
Baseline	9.71 ± 1.66	8.47 ± 1.50	< 0.0001
Final visit	8.02 ± 1.18	6.40 ± 0.43	< 0.0001
Change	−1.69 ± 1.68***	−2.07 ± 1.52***	< 0.0001
**FBG, mmol/l**			
Baseline	10.59 ± 3.11	9.84 ± 2.77	< 0.0001
Final visit	7.27 ± 1.79	6.14 ± 1.03	< 0.0001
Change	−3.33 ± 3.10***	−3.70 ± 2.71***	0.0003
**PPBG breakfast, mmol/l**			
Baseline	15.49 ± 4.08	14.20 ± 3.86	< 0.0001
Final visit	10.06 ± 2.07	8.62 ± 1.81	< 0.0001
Change	−5.43 ± 4.12***	−5.59 ± 3.94***	0.367
**Major hypoglycaemia (events/patient/year)**			
Baseline	0.362 ± 2.30	0.345 ± 1.84	0.325
Final visit	0.018 ± 0.36	0.020 ± 0.32	0.625
**Minor hypoglycaemia (events/patient/year)**			
Baseline	6.82 ± 19.31	8.73 ± 22.94	0.0023
Final visit	1.83 ± 16.39	2.34 ± 8.89	< 0.0001
**Nocturnal hypoglycaemia (minor events/patient/year)**			
Baseline	2.31 ± 10.31	2.78 ± 9.17	< 0.0001
Final visit	0.32 ± 3.22	0.54 ± 3.15	< 0.0001
**Treatment satisfaction (% patients very/extremely satisfied)**			
Baseline	17.6	14.9	0.366
Final visit	52.8	61.0	0.0416
**Prestudy BHI 30 dose (IU/kg)**	0.50 ± 0.25	0.47 ± 0.23	0.0001
**Mean dose of BIAsp 30 (U/kg ± SD)**			
Baseline	0.47 ± 0.22	0.44 ± 0.21	< 0.0001
Final visit	0.48 ± 0.23	0.43 ± 0.19	< 0.0001
Change	0.01 ± 0.17*	−0.02 ± 0.17**	0.0002
**BIAsp 30 bid (% patients)**			
Baseline	85.2	83.9	0.0021
Final visit	85.5	85.6	0.5050

Values are mean ± SD.

* p < 0.01

** p < 0.001

*** p < 0.0001.

BMI, body mass index; BHI 30, biphasic human insulin 30; FBG, fasting blood glucose; PPBG, postprandial blood glucose; BIAsp 30, biphasic insulin aspart 30; bid, twice daily.

The majority of patients who achieved an HbA_1c_ < 7.0% did so without hypoglycaemia (*n*=1483/1489) – major, minor or nocturnal when switching from BHI 30 to BIAsp 30. For these patients, there was a 2.07% reduction in HbA_1c_, from a mean baseline HbA_1c_ of 8.47% to 6.40% at end-point (p < 0.0001) after 6 months of treatment with BIAsp 30.

When switching from BHI 30 to BIAsp 30, significant reductions in HbA_1c_, FBG and PPBG were achieved regardless of whether the dose switch was unit-for-unit, or to a lower or higher dose at the time of transfer (Table 4). However, baseline values of these parameters were higher and reductions at final visit greater in those switching to a higher insulin dose. More patients achieved a target HbA_1c_ < 7.0% when switching unit-for-unit (43.7%) than when switching to a lower (38.5%) or higher dose (32.2%) (Table 4).

**Table 4:** Change in dose from baseline to end-point for three subgroups: patients switching on a unit-for-unit basis, those experiencing a lower dose and those experiencing a dose increase

	Unit-for-unit switchers (*n*=1399)	Patients switching to a lower dose (*n*=1285)	Patients switching to a higher dose (*n*=1172)	Between groups comparison p-value
**HbA**_**1c**_**, %**				
Baseline	9.0 ± 1.6	9.2 ± 1.9	9.4 ± 1.6	< 0.0001
Final visit	7.3 ± 1.2	7.4 ± 1.3	7.4 ± 1.2	0.09
Change	−1.7 ± 1.6***	−1.8 ± 1.7***	−2.0 ± 1.6***	< 0.0001
**% Patients reaching HbA_1c_ < 7%**	43.70%	38.50%	32.20%	0.0011
**FBG, mmol/l**				
Baseline	10.0 ± 3.0	10.2 ± 3.1	10.7 ± 3.1	< 0.0001
Final visit	6.7 ± 1.5	6.8 ± 1.6	6.9 ± 1.8	0.12
Change	−3.3 ± 2.9***	−3.3 ± 2.9***	−3.9 ± 3.0***	< 0.0001
**PPBG breakfast, mmol/l**				
Baseline	14.4 ± 3.9	14.6 ± 4.2	15.7 ± 3.9	< 0.0001
Final visit	9.2 ± 2.1	9.4 ± 2.3	9.7 ± 2.5	0.0005
Change	−5.2 ± 3.8***	−5.2 ± 4.2***	−6.0 ± 4.1***	< 0.0001
**Major hypoglycaemia (events/patient/year)**				
Baseline	0.44	0.25	0.36	0.0004
Final visit	0.04***	< 0.01***	0.01***	0.0048
**Minor hypoglycaemia (events/patient/year)**				
Baseline	8.95	7.84	6.14	< 0.0001
Final visit	2.68***	1.24***	2.11***	0.0002
**Nocturnal hypoglycaemia (minor events/patient/year)**				
Baseline	3.31	2.45	1.84	< 0.0001
Final visit	0.44***	0.22***	0.58***	< 0.0001
**Treatment satisfaction (% patients very/extremely satisfied)**				
Baseline	16.40%	15.30%	16.40%	0.93
Final visit	55.30%	57.40%	60.00%	0.60
**Prestudy BHI 30 dose**				
(IU/kg)	0.49 ± 0.22	0.59 ± 0.26	0.36 ± 0.19	< 0.0001
bid (% patients)	89.30%	87.00%	74.50%	< 0.0001
**Mean dose of BIAsp 30 (U/kg ± SD)**				
Baseline	0.49 ± 0.22	0.39 ± 0.19	0.52 ± 0.2	< 0.0001
Final visit	0.49 ± 0.22	0.43 ± 0.21	0.48 ± 0.2	< 0.0001
Change	0.00 ± 0.17^NS^	0.04 ± 0.17***	−0.03 ± 0.17***	< 0.0001
**BIAsp 30 bid (% patients)**				
Baseline	89.30%	76.30%	87.50%	< 0.0001
Final visit	88.30%	81.90%	86.20%	< 0.0001

Values are mean ± SD.

*** p < 0.0001 vs. baseline, ns = not significant.; BHI 30, biphasic human insulin 30; bid, twice daily; BIAsp 30, biphasic insulin aspart 30; FBG, fasting blood glucose; PPBG, postprandial blood glucose.

### Safety

**Figure 1:**
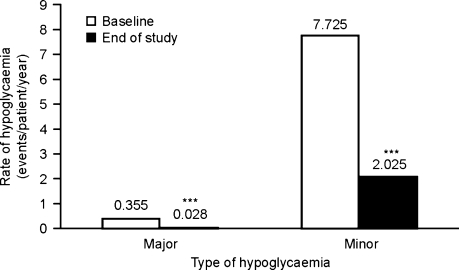
Rates of major (left) and minor (right) hypoglycaemia were lower at the final visit compared with the baseline visit in the main cohort. ***p < 0.0001 vs. baseline

The rate of major hypoglycaemic events reported as SADRs (events/patient/year) was lower at the end of the study when using BIAsp 30 compared with the baseline rate, when BHI 30 was the treatment (p < 0.0001; Table 2, Figure 1). No other SADRs were reported. Minor and nocturnal hypoglycaemia also decreased significantly from baseline at final visit (both p < 0.0001; Table 2, Figure 1).

Major hypoglycaemia rates were similar in patients who reached an HbA_1c_ < 7.0% and in those whose final HbA_1c_ was ≥ 7.0% (0.345 vs. 0.362 events/patient-year; Table 3), while minor and nocturnal hypoglycaemia rates were higher in those reaching this target (minor: 8.73 vs. 6.82 events/patient-year, p = 0.0023; nocturnal: 2.78 vs. 2.31 events/patient-year, p < 0.0001; Table 3).

Major, minor and nocturnal hypoglycaemia rates were significantly reduced from baseline regardless of whether the dose switch from BHI 30 to BIAsp 30 was unit-for-unit, or to a lower or higher dose at the time of transfer (all p < 0.0001; Table 4). Patients who switched unit-for-unit had the highest major and minor hypoglycaemia rates at baseline and final visit (Table 4).

### Weight change

For patients aged over 18 years (*n*=3819 for those supplying baseline and final visit data), there was a statistically significant but minimal weight loss in this subpopulation, from 69.2 ± 13.8 kg at baseline to 69.2 ± 13.4 kg after 6 months (difference from baseline, −0.06 ± 4.86 kg; p = 0.0226). Patients under 18 years were not included in the analyses, as weight gain would have been complicated by growth rate.

Among the dosing subgroups, patients who switched from BHI 30 to BIAsp 30 unit-for-unit gained 0.27 ± 5.56 kg (70.78 ± 15.13 kg at baseline to 71.04 ± 15.17 kg at final visit, p < 0.0001), those who switched to a lower dose had a weight reduction of 0.31 ± 4.12 kg (67.48–67.17 kg, p = 0.0098), and those who switched to a higher dose had no significant weight change: −0.16 ± 4.69 kg (69.31 ± 13.17 kg to 69.15 ± 12.45 kg, p = 0.2272).

### BIAsp 30 dose

At baseline, patients received a mean dose of 31.9 ± 16.7 U (0.46 ± 0.22 U/kg) BIAsp 30. After 3 months, the dose had slightly increased to 32.5 ± 18.4 U (0.47 ± 0.22 U/kg). At the final visit, the mean BIAsp 30 dose was 32.3 ± 18.4 U (0.47 ± 0.22 U/kg), change from baseline: 0.00 ± 0.17. For patients who achieved an HbA_1c_ < 7.0% without hypoglycaemia, total insulin daily dose was 29.6 ± 14.3 U (0.44 ± 0.21 U/kg) at baseline and 28.8 ± 14.2 U (0.43 ± 0.19 U/kg) at final visit (p = 0.0003).

At the point of switching, most patients (*n*=3188, 83.4%) used BIAsp 30 twice daily. The remaining patients used BIAsp 30 once (*n*=459, 12.3%) or three times daily (*n*=160, 4.3%). By the end of the study, the proportion of patients taking BIAsp 30 once, twice or three times daily was 9.3% (*n*=338), 84.7% (*n*=3088) and 5.9% (*n*=216) respectively. Very few patients (*n*=4, 0.1%) used BIAsp 30 four times a day.

In terms of starting regimen, 36.3% of patients switched from BHI 30 to BIAsp 30 on a unit-for-unit basis, 33.3% started BIAsp 30 on a lower dose than BHI 30 and 30.9% upgraded to a higher dose of BIAsp 30 than their previous premixed human insulin. Of these approaches, the unit-for-unit basis incurred the least change in final dose at 6 months (+0.2 U, p = ns) and patients across each subgroup tended to start, and stay, on a twice daily dosing regimen (Table 4).

### Patient satisfaction

When patients were asked to comment on how satisfied they were with their treatment at baseline with BHI 30 and at the end of the study (i.e. following a switch to BIAsp 30), more patients answered ‘very satisfied or extremely satisfied’ at the end of the study: 16.1% (baseline), 56.9% (end of the study) (p < 0.0001 for change from baseline).

## Discussion

Findings from this observational study suggest that patients switching from a human-based premix regimen to a regimen with BIAsp 30 had lower rates of hypoglycaemia with improved glycaemic control, and an increased treatment satisfaction score. A slight weight loss was also observed which, while statistically significant, was deemed clinically irrelevant, and could be explained by the negligible change in insulin dose. These data support the conclusions of a previous observational study with BIAsp 30 (14), which suggested an improved balance between blood glucose control and tolerability for premixed analogues, possibly due to a pharmacodynamic profile better able to mimic the endogenous insulin profile in healthy subjects (7,17). Independent analyses of a cohort of patients who switched from BHI 30 + OAD to BIAsp 30 + OAD and stayed on BIAsp 30 + OAD (data not presented) yielded similar results for the primary end-point, as well as other study measures. This indicates that there was no confounding influence of patients with missing OAD information, or of adding or adjusting OADs or dose during the course of the study on the results.

Ordinarily, part of the value of an observational study is its ability to verify the evidence of clinical trials in a real world setting (18). Here, however, these analyses represent a novel approach to exploring the effects of a particular treatment regimen switch, and as such, can provide valuable insight into how best to manage dose when switching from BHI 30 to BIAsp 30. Patients who switched were divided into three categories: those switching on a unit-for-unit basis, those decreasing their dose and those increasing their dose upon BIAsp 30 initiation. Of these approaches, the first group incurred the smallest subsequent dose increase over the course of the study, with the other two groups increasing and decreasing dose respectively (both p < 0.05). Interestingly, all three groups showed significant improvements in HbA_1c_, FBG and PPBG after 6 months of BIAsp 30 therapy, although a greater proportion of those who switched unit-for-unit achieved the HbA_1c_ target of < 7.0% compared with those who switched to a lower or higher dose. Baseline glycaemic control was also better in the unit-for-unit switchers, so the similar end-of-study results across all groups was achieved by titrating up the dose in those who switched to a lower dose and by titrating down in those who switched to a higher dose.

The final insulin weight-adjusted dose observed here was 0.47 U/kg, which is similar to that found in the China cohort of the PRESENT observational study, but higher than that reported in the India cohort (19,20). While these data must be viewed with the caveat that patients’ therapy should be considered on a case-by-case basis, these findings suggest that switching to BIAsp 30 can be effectively done using a simple unit-for-unit dose switch. Moreover, injection frequency did not change significantly from baseline to end-point for any group, with twice-daily dosing being the most common.

One third of patients were able to reach the American Diabetes Association (ADA) target HbA_1c_ of < 7% (21) without experiencing hypoglycaemia. Interestingly, the mean BIAsp 30 dose decreased by 0.88 U (−0.02 U/kg) from baseline (p = 0.003), suggesting that the significant reduction in blood glucose levels was not achieved by up-titration. This subgroup represented the majority of patients who achieved this HbA_1c_ target at the final visit, highlighting what a barrier hypoglycaemia can be to achieving glycaemic control. Patients in this subgroup had, on average, a lower HbA_1c_ at baseline (8.47% vs. 9.21% for the overall study population), so better glycaemic control and lower BIAsp 30 doses following the switch may have helped these patients stay free from hypoglycaemia. The current recommended threshold for modifying or intensifying a failing diabetes treatment is an HbA_1c_≥ 7% (22). From the present data, switching from BHI 30 to BIAsp 30 provides an effective option for patients.

Whilst our data support the value of optimising a patient’s regimen before glycaemic control diminishes, it is likely that the mean baseline HbA_1c_ of > 9% reflects, a suboptimal control that is common in many populations (23,24). It is therefore reassuring to note that patients with a higher baseline HbA_1c_ also gained significant benefits in glycaemic control and tolerability. This is evident from the results of those patients who reached a target HbA_1c_ < 7.0% compared with those who had HbA_1c_ levels ≥ 7.0% (Table 3). The patients who reached this target had a shorter diabetes duration, lower HbA_1c_ and needed less insulin, suggesting that it is easier to get optimal glycaemic control with BIAsp 30 when prescribing this insulin earlier.

These factors may have contributed to the improved treatment satisfaction scores after switching to BIAsp 30. While an insulin-specific questionnaire such as the Insulin Treatment Satisfaction Questionnaire (25) may have been more appropriate for this cohort, as all patients were previously treated with human premixed insulin, and may possibly have resulted in greater improvements in score, it is encouraging that substantial improvements were achieved with the more general diabetes therapy questionnaire, the DiabMedSat.

Weight changes, although significant in some groups (overall and in patients switching unit-for-unit or to a lower dose) were small and clinically irrelevant – all < 0.5 kg. As expected, patients switching to a lower dose finished on a lower dose than their previous therapy (BHI 30), and so lost 0.31 kg. What is more surprising is that patients who switched to a higher dose and finished on a higher dose than their previous therapy still managed to lose a small amount of weight (−0.16 kg). This may be the result of dietary advice received during physician visits and perhaps a reduced need to ingest sugar due to the significantly lower rates of major, minor and nocturnal hypoglycaemia. These factors may also have had an impact on the observed minimal weight changes in the other subgroups.

As health economic data are best analysed for individual countries, the cost-effectiveness issues of switching patients from BHI 30 to BIAsp 30 have not been addressed in this report on a global cohort. However, results of an analysis based on data from the PRESENT study indicate that switching to BIAsp 30 from BHI 30 in the Chinese setting was associated with increased direct medical costs offset by reduced diabetes-related complication costs over patient lifetimes (26).

While observational studies enable inclusion of a more heterogeneous population due to relaxed inclusion criteria, they have limitations which must be taken into account when evaluating data. Lack of randomisation combined with no comparator or placebo control may undermine the significance of findings, and patient drop-out with missing data may also compromise statistical analyses. Also, clinical practice may vary considerably between countries, clinical centres and individual physicians. Notwithstanding the caveats these factors necessitate, our findings nevertheless show clinically relevant benefits for patients switching to BIAsp 30 from BHI and suggest a straightforward approach to handling dose.

In conclusion, this subanalysis from the IMPROVE™ study supports the positive implications from earlier controlled and observational studies on switching of patients from human premix to BIAsp 30 in routine care. While patients who were switched unit-for-unit or switched to a lower or higher dose all had significant improvements from baseline in glycaemic control and hypoglycaemia at final visit, those who were switched unit-for-unit had the best baseline and end-of-study glycaemic control. These findings support the recently updated European Association for the Study of Diabetes and ADA consensus guidelines for treatment management in type 2 diabetes (22), which state that earlier intervention to improve glycaemic control enables the greatest benefits to patients.
